# Burden of Disease of COVID-19: Strengthening the Collaboration for National Studies

**DOI:** 10.3389/fpubh.2022.907012

**Published:** 2022-06-03

**Authors:** Sara Monteiro Pires, Grant M. A. Wyper, Annelene Wengler, José L. Peñalvo, Romana Haneef, Declan Moran, Sarah Cuschieri, Hernan G. Redondo, Robby De Pauw, Scott A. McDonald, Lynelle Moon, Jad Shedrawy, Elena Pallari, Periklis Charalampous, Brecht Devleesschauwer, Elena Von Der Lippe

**Affiliations:** ^1^National Food Institute, Technical University of Denmark, Kgs Lyngby, Denmark; ^2^Place and Wellbeing Directorate, Public Health Scotland, Glasgow, United Kingdom; ^3^Department of Epidemiology and Health Monitoring, Robert Koch Institute (RKI), Berlin, Germany; ^4^Unit of Non-Communicable Diseases, Department of Public Health, Institute of Tropical Medicine, Antwerp, Belgium; ^5^Département des Maladies Infectieuses, Santé Publique France, Saint-Maurice, France; ^6^School of Public Health, College of Medicine and Health, University College Cork, Cork, Ireland; ^7^Faculty of Medicine and Surgery, University of Malta, Msida, Malta; ^8^Department of Epidemiology and Public Health, Sciensano, Brussels, Belgium; ^9^Department of Rehabilitation Sciences, Faculty of Medicine and Health Sciences, Ghent University, Ghent, Belgium; ^10^Centre for Infectious Disease Control, National Institute for Public Health and the Environment, Bilthoven, Netherlands; ^11^Health Group, Australian Institute of Health and Welfare, Canberra, ACT, Australia; ^12^Department of Global Public Health, Karolinska Institutet (KI), Stockholm, Sweden; ^13^Health Innovation Network, London, United Kingdom; ^14^Department of Public Health, Erasmus Medical Center, Rotterdam, Netherlands; ^15^Department of Translational Physiology, Infectiology and Public Health, Ghent University, Ghent, Belgium

**Keywords:** DALY, disability-adjusted life year, COVID-19, coronavirus, capacity building, European Burden of Disease Network

## Abstract

**Objectives:**

Quantifying the combined impact of morbidity and mortality is a key enabler to assessing the impact of COVID-19 across countries and within countries relative to other diseases, regions, or demographics. Differences in methods, data sources, and definitions of mortality due to COVID-19 may hamper comparisons. We describe efforts to support countries in estimating the national-level burden of COVID-19 using disability-adjusted life years.

**Methods:**

The European Burden of Disease Network developed a consensus methodology, as well as a range of capacity-building activities to support burden of COVID-19 studies. These activities have supported 11 national studies so far, with study periods between January 2020 and December 2021.

**Results:**

National studies dealt with various data gaps and different assumptions were made to face knowledge gaps. Still, they delivered broadly comparable results that allow for interpretation of consistencies, as well as differences in the quantified direct health impact of the pandemic.

**Discussion:**

Harmonized efforts and methodologies have allowed for comparable estimates and communication of results. Future studies should evaluate the impact of interventions, and unravel the indirect health impact of the COVID-19 crisis.

## Introduction

The spread of coronavirus disease 2019 (COVID-19), caused by the severe acute respiratory syndrome coronavirus 2 (SARS-CoV-2), was declared as a pandemic by the World Health Organization (WHO) on the 11th of March 2020 (1). Since the outbreak was first identified in December 2019 in Wuhan, China, the public health and social impact of the disease has evolved to be enormous. It has affected every country, population and person in the world, either directly or indirectly.

Most efforts to understand and compare the health impact of COVID-19 across populations have been made using incidence and mortality-based metrics. However, understanding and quantifying the combined impact of morbidity and mortality is a necessary step to assess both the within-country impact of COVID-19 relative to other causes of disease and injury, in sub-national areas or demographics, and to standardize comparisons between countries. So far, studies of this type have been relatively sparse, presumably due to a mixture of factors, and likely hindered by the need for real-time information, and the lack of analytical capacity and standardized, and robust methods. Summary measures of population health, such as disability-adjusted life years (DALYs), offer a more detailed estimation of the direct impact of the disease in a given population, and can provide future opportunities to assess the indirect impact of the pandemic as a result of preventive measures such as national lockdowns, or of disruption of vital health care services.

The DALY is the key metric in the Global Burden of Disease (GBD) study, a well-resourced and long-standing initiative by the WHO and the Institute for Health Metrics and Evaluation (IHME) (2). The GBD study aims at providing standardized procedures and comparable estimations of a large number of diseases and risk factors across the world. With a narrower scope, local, national, or regional burden of disease studies are useful for quantifying specific populations' health impacts of context-relevant diseases and risk factors, accounting for local characteristics, demographics, and knowledge, and sometimes relying in more granular information complementing GBD's information (3). National burden of disease studies have the advantages of access to country-specific, real-time health and surveillance data, as well as the proximity to local experts on national health systems and public health that are key to the interpretation and usability of the study results. They are also able to involve disease, risk, and methodology experts, as well as facilitate the communication and translation of results to policy making. In Europe, countries such as Belgium, France, Germany, the Netherlands, Scotland, and Sweden, have launched national burden of disease studies in recent years (4–7). Globally, national burden of disease studies (8, 9) and projects dedicated to the burden of specific groups of diseases [for example foodborne diseases (10–14), or infectious diseases more broadly (15)] have also been launched. There is still a pressing need to build capacity in additional countries to estimate the burden of diseases at the national level, and particularly for estimating the burden of new diseases such as COVID-19. As disease burden estimations are dependent on methodological choices such as data collection and metrics used, an international consensus is useful to enhance transparency and produce estimates which are comparable.

The European Burden of Disease Network (*burden-eu*) was established in 2019 to act as a technical platform for integrating and strengthening capacity in burden of disease assessment across Europe and beyond (16). It is structured in technical and disease-focused working groups. At the moment of writing this review (February 2022), the *burden-eu* gathers 330 individual members from 53 countries. Capacity building is one of the key pillars of *burden-eu*, and the ultimate goal of several of its activities. Since the start of the pandemic, *burden-eu* has developed tools and initiatives to support countries in implementing national burden of COVID-19 disease studies. These included developing and harmonizing methodologies, disseminating technical materials, launching a dedicated working group and online discussion forum, exchanging experiences and supporting capacity building, and assistance with the planning of future burden of disease studies.

We reviewed the approach and output of the *burden-eu* to supporting countries in estimating the disease burden caused by COVID-19 at national-level. First, we present the use of the consensus methodology, data input requirements, and solutions to data gaps to estimate national-level burden of COVID-19. At a second step, we provide an overview of national-level studies that have carried out COVID-19 disease burden estimations with study periods between January, 2020 and December, 2021 and conducted across the *burden-eu* countries. Further, we describe the Networks' capacity-building activities and additional actions to address the impact of COVID-19 pandemic by improving data collection and data sharing in the European Union.

## Methods

### Overview of Capacity-Building for National Burden of COVID-19 Studies

In mid-2020, a *burden-eu* working group convened to establish an approach to support the network's members to establish national studies. First, the group discussed the methodology, data requirements, and resources needed to implement a national study. Based on the output of these discussions, a comprehensive protocol was published on the network's website, and a scientific article was published (17, 18). To present this methodology, share already finalized studies, and discuss challenges and opportunities for future studies, the *burden-eu* organized a public webinar, which was attended by over 100 participants in November 2020 (19). The network's website collects and continuously posts all published articles related to the burden of COVID-19 (20). The *burden-eu* also formed the Burden of COVID-19 Task Force, which is open to all network members conducting or interested in implementing national studies. This task force aims to share experiences in national burden of COVID-19 studies; support each other with disease burden calculations, model assumptions and data gaps; harmonize methodologies and align strategies for communicating results; and discuss research and upcoming evidence on long-COVID. The group meets regularly to work toward achieving these aims. Lastly, the *burden-eu* launched an online discussion forum, where members can post questions and receive answers from peers in an interactive and rapid way.

### Consensus Methodology to Estimate the Direct Burden of COVID-19

**Figure 1:**
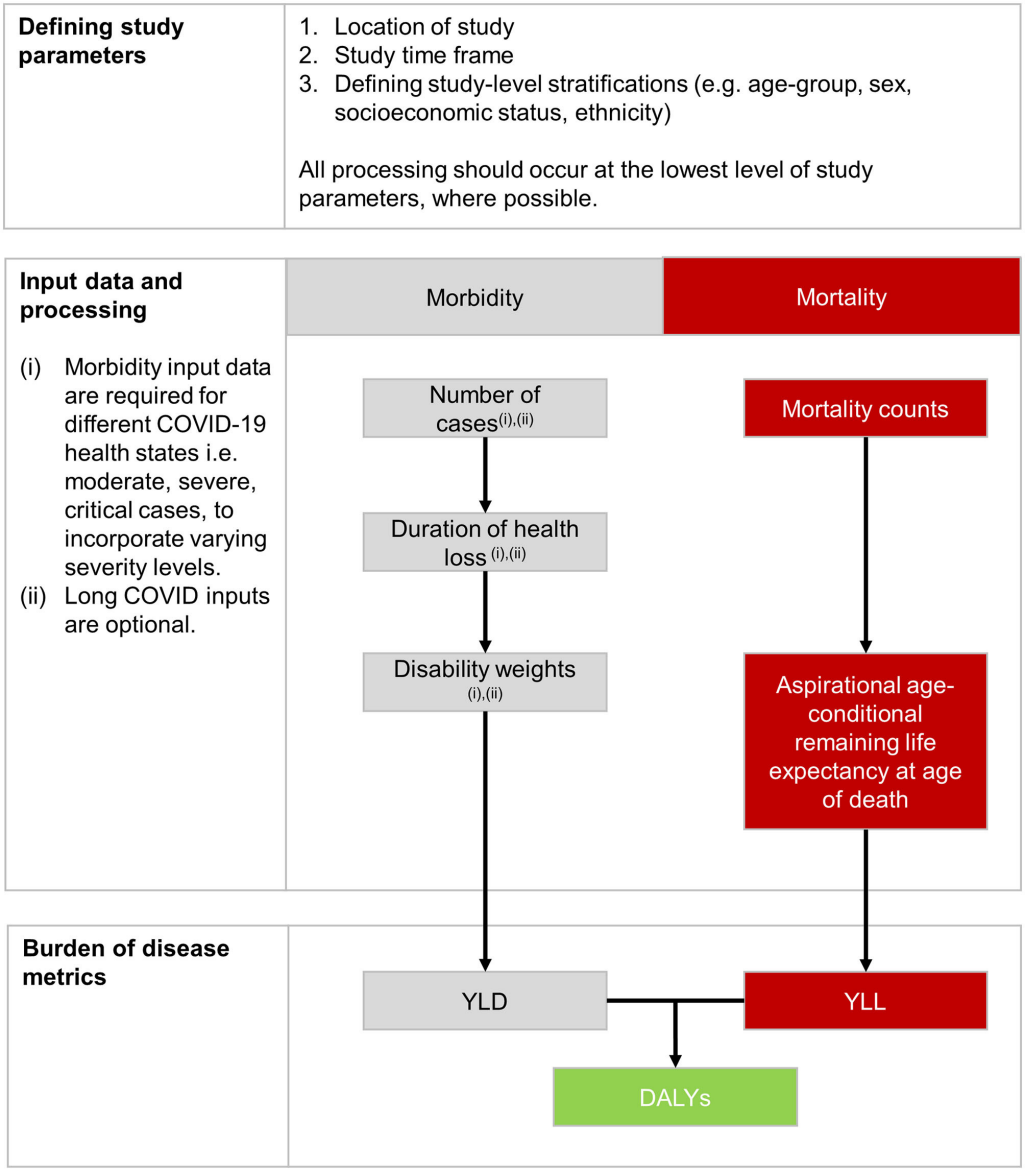
Steps and processes for the calculation of disability adjusted life years (DALYs) of COVID-19 at national level.

The protocol to estimate the burden of COVID-19 at national level specified data requirements, reflecting the data availability and quality of data inputs by country, offered solutions to overcome data gaps, and a consensus approach for calculations (17). It presented the approach in three steps: defining study parameters; estimating the impact of morbidity, in terms of years lived with disability (YLD); and estimating the impact of mortality, in terms of years lost to premature death (YLL) (Figure 1).

#### Defining Study Parameters

DALYs quantify the full population health impact and are calculated by summing YLD and YLL. DALYs can be estimated based on grouped characteristics of interest, such as demographics (e.g., age, sex, socioeconomic status, and ethnicity), geographical region, or time.

#### Estimating Years Lived With Disability

Diseases caused by an infectious agent may consist of one or more health outcomes, which can have acute and/or chronic phases, with varied durations. A major methodological choice for calculating YLDs is whether to use an incidence- or prevalence-based approach. An incidence-based approach has been considered the most suitable approach for estimating the burden of infectious diseases (21). In this approach, all health outcomes and the associated disease burden, including those health outcomes occurring long after the acute infection, are assigned to the initial event, i.e., the infection with the agent (22). A wide range of acute symptoms of COVID-19 have been reported, ranging from mild to severe respiratory symptoms; the latter often leading to hospitalization and intensive care (23). Wyper et al. (17) proposed a disease model defining the direct health outcomes of COVID-19, which can be adapted to reflect the data available in each country and evidence that becomes available with time. For example, the model should be adapted to reflect differences in restrictions and triaging across hospitals, regions, and countries, and how the state of the pandemic increases the pressure on the healthcare system, affecting the quality and access to its services. This emphasizes that the distribution of disease incidence across health states is likely to vary by location, as prevention and control strategies have varied during the pandemic (24).

In addition to acute symptoms, a proportion of patients have reported long-lasting symptoms of COVID-19, a multi-faceted condition referred to as post-acute sequelae, post-acute COVID-19 syndrome, or “long-COVID” (25, 26). At the time of writing this review, information on the incidence of the various manifestations of long-COVID and related health outcomes, as well as their duration and severity, were still sparse. Thus, only a few studies have included long-COVID in burden of disease assessments, and the ones that did relied on assumptions and simplifications to overcome data gaps (27–29). Several cohort studies have been launched globally to collect such data; once these data are available, burden of disease estimations can be updated to provide more complete and accurate results of the burden of COVID-19 at national and global levels. The development of an internationally recognized standard definition for long-COVID would greatly facilitate this process (30). In addition, it may be relevant to consider the further development of more granular disability weights. At present, there is a single disability weight to capture post-acute consequences of acute infection, and given the range of symptoms reported for COVID-19, a mild, moderate and severe characterization of disability weights would be a useful development.

#### Estimating Years of Life Lost

When estimating YLL, two inputs are required: the number of deaths; and normative life expectancy. The use of an aspirational life table is the gold standard approach for calculating YLL, as it ensures internationally comparable results, which are particularly important during a public health emergency of international concern (31). It is also essential that YLL is not adjusted for the differences in life expectancy between persons with or without comorbidities. A number of researchers have indicated that such methods are required, but doing so would remove the ability to make comparisons with other diseases or injuries, between different countries, or across time periods. Furthermore, these adjustments could lead to unethical outcomes if the application of public health interventions are guided by YLL, as disadvantaged regions would suffer the most if YLL is adjusted for comorbidities or for lifestyle risk factors; these regions would be deemed to have “less to gain” compared with regions with a lower prevalence of the same comorbidities and/or lifestyle risk factors.

Estimates of the number of deaths will largely depend on data availability. Some countries may have timely mortality data available by mutually exclusive causes of death, in which case the underlying cause of death should be used. Depending on the national death registration policy, some death certificates may indicate ill-defined causes of death. If possible, then redistribution can be considered to refine estimates, in line with methods used for allocating ill-defined deaths to non-COVID-19 causes of death. The definition of COVID-19 deaths may also be a challenge, and vary between countries, despite the standard definition proposed by the WHO (32). Where data allow for estimates to be calculated according to different definitions, it would be preferable to include these as scenario analyses. Doing this would allow for some additional triangulation when interpreting estimates, and may help to provide a bridge between studies that have only have access to a certain mortality definition. The WHO proposed the definition of a death due to COVID-19 as “a death resulting from a clinically compatible illness, in a probable or confirmed COVID-19 case, unless there is a clear alternative cause of death that cannot be related to COVID-19 (e.g., trauma) (32).”

## Results

### Burden of Disease of COVID-19 in European Countries and Beyond

COVID-19 DALY estimates have been published for various countries within the *burden-eu*. Up to the time of finalizing this review [14th February, 2022], burden of disease estimates from Australia, Denmark, Germany, Ireland, Malta, the Netherlands, Scotland have been published (Table 1); Belgium and France are finalizing their estimates for publication, whilst Cyprus and Sweden have embarked upon data collection for their national study. The methods used are aligned with the guidelines developed by *burden-eu* for estimating DALYs due to COVID-19.

**Table 1:** Overview of published and ongoing National Burden of COVID-19 studies, methodological characteristics, and main results published between January 2020 and December 2021.

**Country**	**Period of analysis**	**Estimation of total symptomatic infected**	**Reference life expectancy table**	**Data source for mortality due to COVID-19**	**Long-COVID included**	**DALY/100,000**	**% YLD**	**References**
Australia^a^	1 Jan−31 Dec 2020	No (notified positives only)	GBD-2010	National death registrations (Australian Bureau of Statistics data)	Yes, estimated	32.7	3.5%	(28)
Belgium	Mar 2020–31 Dec 2021	Yes (SEIR modeling)	GBD-2019	Sciensano COVID-19 mortality registry (confirmed +suspected)	Yes	N/A	N/A	Ongoing
Cyprus	9 March 2020–8 March 2021	No (notified positives only)	GBD-2019	Health Monitoring Unit (Cyprus Ministry of Health)	N/A	N/A	N/A	Ongoing
Denmark	28 Feb 2020–28 Feb 2021	Yes (based on survey of the population)	GBD-2019	Death registry	No	520	1.6%	Under review
France		Yes (notified positive cases only)	GBD-2019	National mortality database, i.e., CépiDC (included both confirmed +suspected)	Yes, limited	N/A	N/A	Ongoing
Germany	1 Jan−31 Dec 2020	No (notified positives only)	Germany 2016/2018	Notifiable cases of COVID-19	No	368	0.7%	(33)
Ireland		Notified positives with estimation of asymptomatic cases	GBD-2019	Central Statistics Office, Ireland.	Yes, estimated	1,033	1.3%	Under review
Malta	7 Mar 2020–31 Mar 2021	Yes (notified positives adjusted for under ascertainment)	GBD-2019	Daily COVID-19 bulletins issued by Malta Ministry of Health	Yes, limited	1,086^c^	5%	(27)
Netherlands	1 Jan−31 Dec 2020	Yes (evidence synthesis)^b^	GBD-2019	Statistics Netherlands registered (confirmed +suspected)	No	1,570	1%	Under review
Scotland (29)	1 Jan−31 Dec 2020	Yes (SEIR modeling)	GBD-2019	Death registry (confirmed only or confirmed +suspected)	Yes, limited	1,770–1,980	2%	(29)
Sweden	Mar 2020–Dec 2021	No (notified positive cases only)	GBD-2019	Cause of death register, The National Board of Health and Welfare	Yes, based on national data	N/A	N/A	Ongoing

a *The Australian Burden of Disease Study liaised with burden-eu's Burden of COVID-19 Task Force to ensure methodological harmonization*.

b *For the Netherlands, “symptomatic” is defined using the ECDC case definition, and total symptomatic infected is estimated by synthesizing evidence from a population-level seroprevalence survey, notified cases, and age-group specific estimates of case-ascertainment and the proportion of infections that are symptomatic*.

c *Calculated from the reported estimate of 5,478 DALY and a population size of 505,200 (World Bank estimate for 2019)*.

*DALY, disability adjusted life year; YLD, years lost due to disability; GBD, global Burden of disease; SEIR, susceptible-exposed-infectious-removed*.

Table 1 maps national-level COVID-19 burden of disease studies undertaken across European countries and beyond over the period between January 2020 and December 2021. Currently available estimates show that the direct burden of COVID-19 has varied across countries, but that the contribution of YLL (i.e., of mortality) to the overall burden has been consistently high (between 95 and 99%). Other studies, not supported by *burden-eu*, have estimated DALYs of COVID-19 in countries globally, for example in India, Iran, Mexico and Korea (34–37). Aligned with the findings of studies here presented, the estimated burden of disease varied across countries, but the contribution of YLL to overall DALY was large.

### Country-Specific Adaptations

To adjust to the type and extent of data available and to overcome data gaps, countries embarking on these disease burden analyses made adaptations to the proposed approaches. Describing these methodological choices is important for well-informed comparisons and for knowledge translation at national and international levels. For example, some studies expanded upon the core health states defined by the consensus model. Scotland, Malta, Ireland, France, and Australia included estimates of post-acute consequences following the acute infection period, based on published transition probabilities and durations. Germany was the only country to define mild cases in YLD calculations. The Netherlands employed adjustment factors to correct for underreporting of hospital admissions and deaths. It was recognized that notified cases greatly underestimated the true incidence of infections, particularly in the first period of the pandemic, and thus evidence from seroprevalence survey data, case-ascertainment, and other sources were synthesized to estimate the cumulative incidence of symptomatic SARS-CoV-2 infection. There were also variations in how the duration of health states were defined. Most countries used durations derived from their national data collections. Ireland used the GBD 2019 duration for lower respiratory infections, due to a lack of national evidence on the duration at each health state level. Scotland used daily prevalence estimates from a Susceptible-Exposed-Infectious-Removed (SEIR) transmission model, and daily hospital prevalence data, so did not require any definition of duration. All studies used the GBD aspirational life expectancy life table to value the loss of life, with the exception of Germany that employed the highest observed age-specific residual life expectancy among all the German federal states based on the 2016/2018 life tables.

### International Efforts to Collect and Share Data for Evidence-Based Response to COVID-19

The rapid and wide spread of COVID-19 cases, together with the efforts of countries and international health agencies to monitor the disease, and the overflow of patients to medical care have generated a wealth that can help to fully characterize the epidemiology and clinical aspects this new disease. The emergency of pandemic has introduced an unprecedented response by the research community in demand of collaborative data sharing networks to enrich and accelerate informed decision making. International collaboration to collect, store, and manage relevant health and epidemiological data regarding COVID-19 helps filling data gaps and facilitates robust estimations, including burden of disease metrics, that can help and policy evaluation. In response to the emergency calls launched by the European Commission in May 2020, the *unCoVer* (Unraveling Data for Rapid Evidence-Based Response to COVID-19) project was sponsored as a Coordinated Support Action. The *unCoVer* is defined as a functional network of 29 partners that was established to bring together European and international expertise to monitor, identify, and facilitate the access and utilization of COVID-19 patient's data, to identify knowledge gaps, underrepresented populations, and proactively seek synergies with complementary clinical databases. The *unCoVer*'s members are capable of collecting and utilizing data derived from the response and provision of care to COVID-19 patients by health systems across Europe and internationally. The data within the network comprise mostly information from electronic medical records from hospitals, but also national surveillance data, and registries, and is reached through a federated data infrastructure that ensures data protection and ethical and legal compliance. Thus far, they integrate information from over 20 databases and a sizeable number of COVID-19 patients, which is anticipated to increase as databases are being continuously updated (38). These data may inform future burden of disease assessments, by gaining a deeper understanding on the disease model and variations among heterogeneous groups of patients, including COVID-19 manifestations in vulnerable population subgroups, and shedding light into post-acute COVID-19 conditions that may add to the YLD component of the DALY.

The scientific network “BoCO-19—The Burden of Disease due to COVID-19” was launched in May 2021, coordinated by the Robert Koch Institute in Germany and is funded for a period of two years (39). The overall aim of BoCO-19 is to harmonize burden of disease methodology for the surveillance of dynamic outbreaks, using COVID-19 as an example. The BoCO-19 has established a functional network of partner institutions from countries from the South-East Europe, Southern Caucasus and Central Asia. The *burden-eu* serves as an additional project partner, by contribution with the output of discussion of methodologies for measuring the disease burden of COVID-19. The vision of the BoCO-19 project is to provide a scientific a platform for intensive knowledge exchange and application of a consistent methodology considering context-specific conditions toward calculating the burden of the COVID-19 disease in the pandemic monitoring stage. Efforts are also focused on the dissemination of the harmonized methodology and estimates of the disease burden indicators to the wider scientific community.

## Discussion

The initial capacity building activities have allowed national studies to get off the ground and produce initial sets of results. While burden of COVID-19 studies dealt with various data gaps and a number of assumptions made in the face of knowledge gaps, they delivered broadly comparable results that allow for an interpretation of consistencies, as well as differences in the quantified direct health impact of the first year of the pandemic. Furthermore, whilst many experienced with national burden of disease studies have been among those carrying out COVID-19 DALY studies, some countries have had unintended benefits of their COVID-19 studies, which have led to novel advances in their projects. In particular, the French and Irish studies have now launched their independent DALY studies, which can be augmented with future non-COVID-19 assessments.

At first glance, it can seem challenging to validly compare DALY COVID-19 estimates given difference in underlying data sources, data collection systems, degree of ascertainment of the true incidence of infection by reported cases, disability durations, and definitions of mortality due to COVID-19. Close adherence to the aforementioned protocol (17, 18) by several of the national studies has greatly facilitated comparisons of COVID-19 burden across countries. Given that YLL—which is dependent on complete and accurate recording of death due to COVID-19—accounts for the vast majority of the disease burden, attention to cause of death definitions and addressing under-reporting in COVID-19 deaths will have the most impact on the validity of national estimates, and thus also the comparability of estimates.

These findings illustrate how burden of disease indicators, and standardization of approaches where applicable, can be useful for monitoring within- and across-country public health in an ongoing pandemic. As the push for additional countries and regions to follow continues, our efforts to assist countries to translate their results in a within- and across-country setting will also continue through our Knowledge Translation Working Group. These activities will allow for users to see that collaborative benefits can have both local and wider benefits.

At the onset of the pandemic, we assessed the levels of vulnerability to severe outcomes from COVID-19 infection across Europe (40). This indicated that increasing pre-existing levels of vulnerability were likely to lead to inequalities in adverse outcomes due to the differences in demographic construct and population health levels within individual countries. This means that, even when standardizing results, interpretation of the success or failure between comparisons is not always clear cut, because the level of threat faced by individual countries was unequal. Integrating the impact of inequality both within- and across-countries is important as we progress our work. Evidence has indicated that socioeconomic inequality-attributable COVID-19 DALYs are 40% in Scotland, a result which has been borne out of a legacy of systemic inequality (41). As the pandemic has evolved, monitoring inequalities within countries will give indications into how successful attempts have been to mitigate inequalities, e.g., by prioritizing certain groups for vaccination before others. Communicating these results are important for national and local policy-makers, to scale the size of challenges faced on the public health and health and social care systems of a country.

When our activities began, there was no vaccination for COVID-19. While it is certainly not sufficient alone, vaccination remains the primary tool in preventing deaths and severe illness from COVID-19. Previous evidence has indicated the extent of deaths averted through vaccination in countries of Europe (42). Through continuous monitoring of COVID-19 DALYs, an area of interest will be to start to estimate the DALYs averted through vaccination programmes.

Furthermore, efforts thus far have mainly focused on the direct impact of COVID-19. Future studies need to unravel the indirect health impact of the COVID-19 crisis, linked, e.g., to delayed health care use, increases in domestic violence, or decreases in road traffic accidents. Due to its comprehensive nature, the DALY metric would be well-suited to estimate the overall health impact of the crisis, combining both positive and negative health effects, over a wide range of health outcomes. In the near future, we will continue working toward more complete characterization of the risks, severities, and duration of health outcomes, comprising “Long-COVID.”

## Author Contributions

SP, GW, AW, JP, and EV designed the review and wrote the first version of the manuscript. All authors contributed to the review and provided input to the text. All authors contributed to the article and approved the submitted version.

## Conflict of Interest

The authors declare that the research was conducted in the absence of any commercial or financial relationships that could be construed as a potential conflict of interest.

## Publisher's Note

All claims expressed in this article are solely those of the authors and do not necessarily represent those of their affiliated organizations, or those of the publisher, the editors and the reviewers. Any product that may be evaluated in this article, or claim that may be made by its manufacturer, is not guaranteed or endorsed by the publisher.
